# The Effect of Action Observation Combined with Motor Imagery Training on Upper Extremity Function and Corticospinal Excitability in Stroke Patients: A Randomized Controlled Trial

**DOI:** 10.3390/ijerph191912048

**Published:** 2022-09-23

**Authors:** Jong-Bae Choi, Seo-Won Yang, Sung-Ryong Ma

**Affiliations:** 1 Department of Occupational Therapy, Sangji University, 83 Sangjidae-gil, Wonju-si 26339, Korea; 2 Department of Health Sciences, Hallym University, 1 Hallymdaehak-gil, Chuncheon-si 24252, Korea; 3 Department of Occupational Therapy, Chosun University, 309 Pilmun-daero, Dong-gu, Gwangju 61452, Korea

**Keywords:** stroke, action observation, motor imagery training, upper extremity function, corticospinal excitability

## Abstract

This study aimed to investigate the effect of motor imagery (MI) intervention with action observation (AO) on upper extremity function and corticospinal activation in stroke patients. MI and AO are two forms of motion simulation that activate the motor system without physical activity. There are many studies that show the effect of AO and MI alone, but there are few studies in parallel. This study was conducted on 45 patients who were divided into an experimental group (*n* = 22) and a control group (*n* = 23) using a computer randomization program. The experimental group conducted AO with MI, and the control group only AO. All participants received interventions for twenty-five minutes per session, five times a week, for eight weeks. For the pre- and post-evaluation of all participants, motor evoked potential (MEP) amplitude was measured to compare corticospinal activation, and Fugl-Meyer Assessment Upper Extremity (FMA UE), Wolf Motor Function Test (WMFT) and Motor Activity Log (MAL) were evaluated for changes in upper extremity function. In comparing the amount of change before and after the intervention, a significant change was observed in the experimental group’s MEP amplitude. In the comparison between groups after the intervention and the amount of change before and after the intervention, the experimental group showed significant changes in FMA UE and MAL Amount of Use (MAL AOU) items. As a result of this study, AO with MI is effective in enhancing upper extremity function and increasing cortical spinal cord activation in patients with severe stroke with limited movement.

## 1. Introduction

Stroke is the leading cause of disability in millions of people worldwide, and the patient population will inevitably increase as life expectancy increases [1]. Impairment of motor function is a common sequela of stroke, and hand function is impaired in more than 85% of acute stage patients. Muscle weakness, stiffness, and limitation of joint range of motion in the injured upper extremity act as factors that negatively affect daily functional activities [2]. In addition, restrictions in upper extremity movements, such as reaching, grasping, and manipulating, affect basic daily activities such as personal hygiene, eating, and dressing, leading to decreased quality of life [3]. Human hand use plays an important role in most social and cognitive functions, including performing most activities of daily living and occupational activities. Hand motor skills or the ability to solve tasks accurately, quickly, and reasonably affect survival. This technique achieves purposeful movements in human beings. As the complexity of hand movements increases, the interaction of the central nervous system increases to support effective functional tasks [4,5]. Therefore, restoring upper extremity function in stroke patients can be an important factor in improving their daily life performance. Neurorehabilitation after stroke aims to improve limited movement using neuroplastic effects, usually through repetitive physical training, constraint-induced movement therapy or neurodevelopmental therapy [6,7]. Some useful interventions highlighted in previous studies include electrical stimulation therapy, bilateral hand training, biofeedback, and robotic interactions [8,9]. However, given that upper extremity movement can be significantly impaired in the acute stage after stroke, various interventions may not be appropriate if there is no physical activity at all [10,11]. Motor imagery (MI) and action observation (AO), which have been proposed as alternative treatments for impairment of upper extremity function, can achieve functional improvement in physical activity and are proposed as viable and valuable interventions for physical rehabilitation training [12].

MI and AO are two motion simulation forms that activate the motor system without motor execution. AO is thought to be a bottom-up process that communicates the observed behavior to the sensorimotor system of the observer, allowing the behavior to be understood [13]. However, MI has been modeled as a top-down process that reflects the inner movement rehearsal process through efferent information of action [14]. Therefore, both AO and MI interact with internal representations of the body and behavior, but from different perspectives. MI is a type of mental training involving the internal generation of movement’s visual and kinesthetic aspects. Many studies have reported that therapists aiming at motor learning and rehabilitation should use MI to improve motor skills [15,16]. It can be used as a pre-stage of body movement to improve functional activity or as an important alternative intervention when movement is restricted owing to neurological damage or injury [14]. MI training can improve motor performance in neurorehabilitation, and neural reconstruction after MI training is similar to the changes after physical training [13,15]. Corticospinal activation was reported in a functional magnetic resonance imaging (fMRI) study of neuroplastic effects associated with MI training in stroke patients [17]. In addition, it was reported that AO induces active body movements by internal movements stored through observed movements [18]. Therefore, AO has been recommended as a neurorehabilitation treatment. Furthermore, previous studies reported a significant increase in neurophysiological activity observed in the premotor area of the cortex using fMRI [19]. This increase was particularly pronounced in stroke patients assigned to the AO therapy group compared to controls. Brain regions that exhibit neuroplastic effects include the bilateral ventral premotor cortex, bilateral superior temporal gyrus, supplementary motor regions, and the contralateral marginal gyrus. Therefore, the authors argued that the ability of mirror neuron systems to reproduce observed behaviors should be used to facilitate rehabilitation and neuroplastic effects in motor areas of the brain [20]. In general, studies to investigate the effects of two types of motor simulations, AO and MI, on stroke patients were conducted independently or in comparison [17]. Recently, a study examined the potential benefits of combined intervention of AO and MI (AO + MI). It provided positive and consistent evidence showing that AO + MI can significantly increase the level of neurophysiological activation in the cortical-motor regions of the brain [21,22]. However, it was mostly a study of healthy subjects, a pilot study of a small number of subjects, and a study combining AO and MI of simple body movements. Therefore, this study aimed to investigate the effect of MI intervention with AO on upper extremity function and corticospinal activation in subacute stroke patients using a study design that supplements the limitations of previous studies.

## 2. Materials and Methods

### 2.1. Participants

The participants in this study were 45 patients admitted to hospital A in Seoul and hospital B in Gyeonggi-do who were diagnosed with stroke and were receiving rehabilitation treatment. All study participants were those who listened to detailed explanations about the purpose and experimental method of the study and expressed their intention to participate voluntarily in the study. The inclusion criteria were as follows [21]: diagnosed with stroke by a neurologist and subacute patients with an onset period of 2 to 8 months, having a score of 24 or higher in the Mini-Mental State Examination—Korean, presence of an upper limb MMT fair or less on the paralyzed side, absence of visual impairment or unilateral neglect, and having an average score of 2.26 or less in the Vividness of Movement Imagery Questionnaire test [23]. The exclusion criteria were as follows: the presence of an attached artificial pacemaker, the presence of a metal implant in the brain, the use of antipsychotic drugs, and the use of drugs related to spasticity.

### 2.2. Procedure

**Figure 1:**
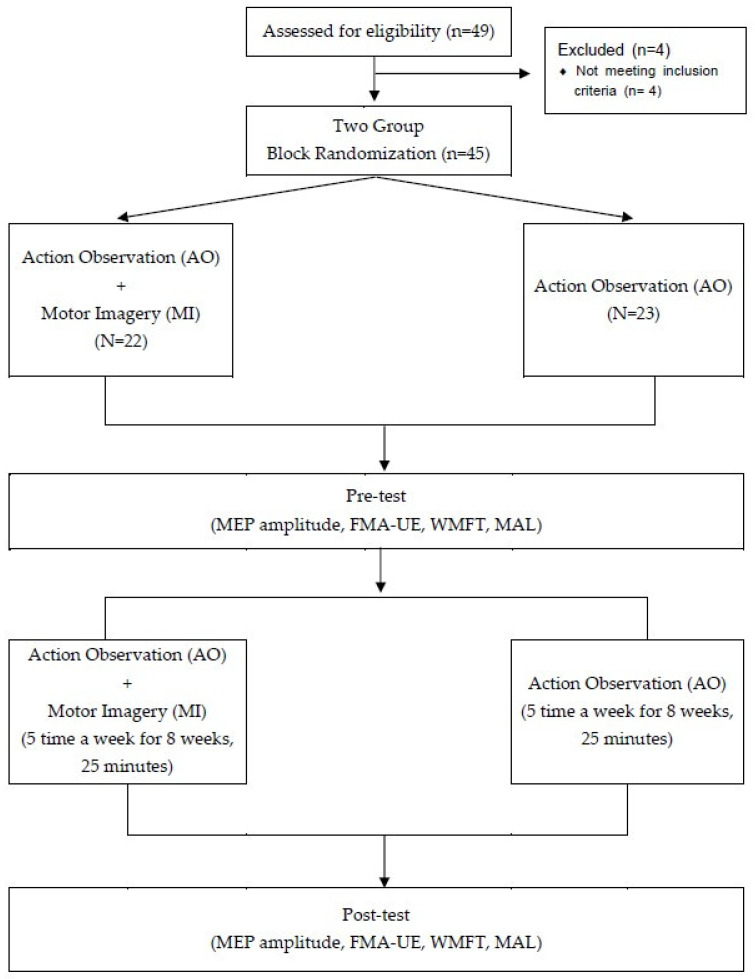
Flow diagram of the study.

This study was conducted from May to December 2020, and the 45 patients were divided into an experimental group (*n* = 22) and a control group (*n* = 23) using a computer randomization program to participate in the study. In this study, the experimental group performed AO with MI, and the control group performed only AO. The study was conducted by occupational therapists with more than 10 years of clinical experience, and all participants received interventions for 25 min per session, 5 times a week, for 8 weeks. All subjects received additional occupational therapy and physical therapy for 30 min per day. For the pre- and post-evaluation of all participants, motor evoked potential (MEP) amplitude was measured to compare corticospinal activation, and Fugl-Meyer Assessment Upper Extremity (FMA UE), Wolf Motor Function Test (WMFT), and Motor Activity Log (MAL) were evaluated for changes in upper extremity function (Figure 1).

### 2.3. Intervention

#### 2.3.1. AO in Parallel with MI

**Figure 2:**
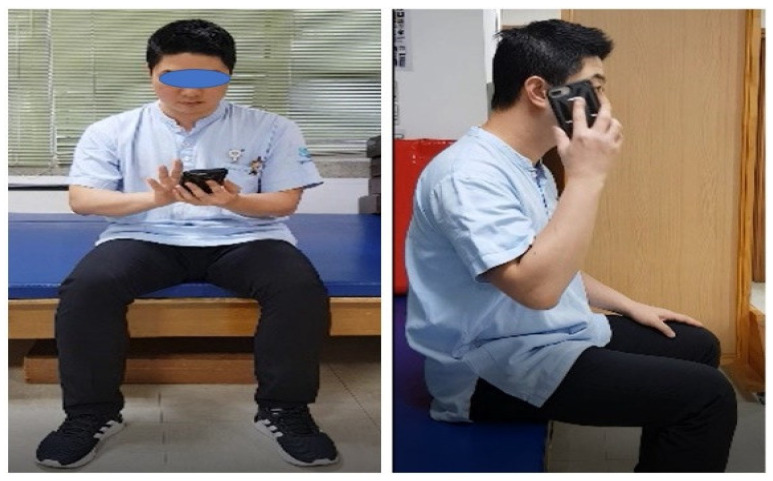
One of ten activities in AO + MI interventions. (ex: using a mobile phone).

The experimental group conducted AO + MI, and among the ten basic daily life activities tasks, five purposeful and meaningful activities the participants wanted to perform were selected and performed. The 10 tasks were as follows [24]: (1) using chopsticks, (2) using a pencil, (3) using a computer mouse, (4) hand washing, (5) using a mobile phone, (6) putting on clothes, (7) drinking with a water bottle, (8) grasping and releasing a tennis ball, (9) handling of a credit card, and (10) combing hair (Figure 2).

**Figure 3:**
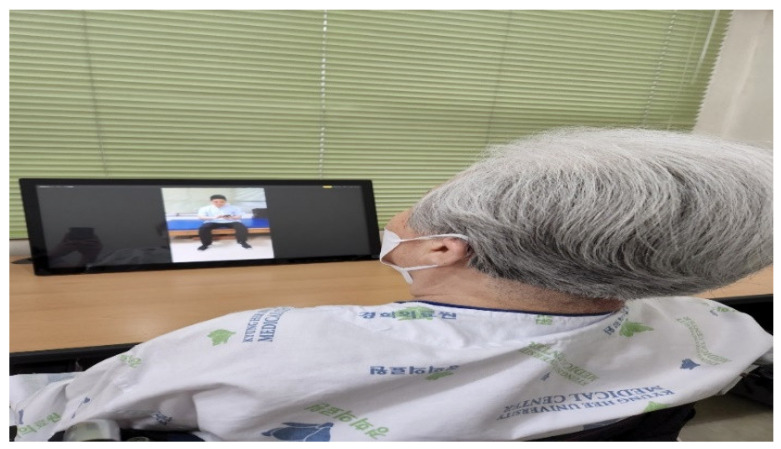
AO + MI intervention.

The 5 selected tasks were carried out for a total of 25 min, and 1 was performed for 4 min with a rest time of 1 min. The 10 tasks were selected by referring to the tasks whose effects were verified in previous studies. Among them, the images performed by the therapist or guardian were divided into front and side images for five tasks to be selected by the patient from a first-person perspective [15]. This was performed using a 15-inch laptop placed on a 70 cm high table in an independent space in a quiet environment. For each task of 4 min, AO and MI are performed simultaneously rather than divided in time. The therapist assumed that the real person in the video was him and instructed them to imagine how their upper extremities and hands would move, concentrating on observing them with their eyes (Figure 3).

#### 2.3.2. AO

In this study, the AO conducted in the control group was performed under the same conditions and methods as the AO + MI conducted in the experimental group. However, the participants were instructed to do only AO using video for their selected five tasks.

### 2.4. Outcome Measures

#### 2.4.1. Motor Evoked Potential (MEP) Amplitude

The MEP amplitude used in this study was a Nicolet Viasys Viking Select EMG EP system (San Diego, CA, USA). MEP is an electrodiagnostic test that induces peripheral muscle responses by directly inducing transcranial magnetic stimulation to the cerebral motor cortex [25]. Magnetic stimulation was performed by placing the central part of the coil stimulator in the Cz position using the International EEG 10–20 recording method. The target muscle was first dorsal interosseous (FDI), and magnetic stimulation was applied to the motor cortex at an angle of 45 degrees from the center of the skull [26]. The point at which the maximum response occurred was determined by moving in small increments. The maximum magnetic field strength was 2.0 Tesla, and the stimulation time was 0.1 ms. The stimulus intensity gradually increased from 80% to 100%. Electromyography values were measured by attaching a silver chloride electrode to the FDI of the injured hand and attaching a ground electrode to the front of the forearm. The resting motor threshold was defined as the minimum stimulation intensity at which MEP > 50 μV was recorded five or more times during 10 stimulations. The amplitude of MEP was determined by measuring the amplitude 10 times after stimulation at 120% [27]. The peak-to-peak amplitude of MEP induced in the contralateral target muscle was obtained [25].

#### 2.4.2. Fugl-Meyer Assessment Upper Extremity (FMA UE)

FMA UE is an evaluation tool for overall motor function in stroke hemiplegic patients. It is a comprehensive evaluation tool that quantitatively measures motor function, balance, sensation, joint range of motion, and pain [28]. In this study, only items related to upper extremity motor function evaluation were selected to examine changes in upper extremity function. With a total of 33 questions, the paraplegic and non-paraplegic sides were each performed thrice, a high score was adopted, and 0~2 points were given depending on the degree of performance. Scores were divided into 0 points for ‘not performing’, 1 for ‘partially performing’, and 2 for ‘completely performing’. The total score of evaluation items for the upper extremity motor function was 66 points, and the higher the score, the higher the level of exercise recovery. The intra-inspector reliability of this evaluation tool is 0.99, the inter-inspector reliability is 0.98, and the test-retest reliability is 0.94 [29].

#### 2.4.3. Wolf Motor Function Test (WMFT)

Wolf developed WMFT in 1989 to evaluate upper extremity motor function in hemiplegic patients. The exercise performance and performance time of each activity are measured as a score, and WMFT consists of 17 tasks ranging from simple to complex movements. The score is given on a 6-point scale from 0 to 5, where 0 means ‘not performed’ and 5 means ‘normal movement’. The inter-rater reliability of the function score of this tool was 0.88, and the inter-rater reliability of the performance time was 0.97 [30].

#### 2.4.4. Motor Activity Log (MAL)

The MAL evaluation tool is divided into a quantitative evaluation tool to determine how often the injured upper limb of stroke patients is used in performing daily activities and a qualitative evaluation tool to determine how well the injured upper limb is used. As an evaluation tool that can be evaluated, it is a structured interview-type evaluation tool [31]. This evaluation tool consists of 30 items related to daily life behavior and is evaluated by dividing it into Amount of Use (AOU) and Quality of Movement (QOM). The evaluation score for each item is given on a 6-point scale from 0 to 5, and the score of each item is summed up and expressed as a total score. The average score is calculated by dividing by the number of evaluation items. The higher the average score, the higher the level of upper extremity function is judged. The internal consistency of this evaluation tool is Conbach’s α = 0.88~0.95, the inter-death reliability is 0.91, and the test-retest reliability is 0.94, making it a highly reliable evaluation tool [32].

### 2.5. Statistical Analysis

The data collected in this study were analyzed using the SPSS Version 21.0 program. The homogeneity was verified using the frequency analysis of descriptive statistics and the chi-square test to analyze the participants’ general characteristics. A paired *t*-test was used to examine the changes before and after intervention in the experimental and control groups, and an independent *t*-test was performed to determine the difference between the two groups. In addition, an independent *t*-test was performed to compare the amount of change before and after the experiment between the two groups. Statistical significance was set at *p* = 0.05.

### 2.6. Ethical Approval

The study was conducted in accordance with the guidelines of the Declaration of Helsinki and was approved by Institutional Review Board (Seoul, Korea) (2020-10-002-001). The study’s aims and the protection of participants’ privacy were fully explained. Once the participants agreed to participate in the study, they signed an informed consent form before the beginning of the study.

## 3. Results

### 3.1. Participants’ Characteristics

The general characteristics of the 45 participants are shown in Table 1. The homogeneity test between the two groups showed no significant difference in all items.

### 3.2. Upper Extremity Function Evaluation

In the comparison before and after the intervention within the groups, both the experimental and control groups showed significant changes in all upper extremity function evaluation items. In comparison between groups after intervention, significant changes were observed in FMA UE (*p* = 0.002) and MAL AOU (*p* = 0.022) items in the experimental group.

In comparison of the amount of change before and after the intervention, significant changes were observed in FMA UE (*p* = 0.000) and MAL AOU (*p* = 0.000) items in the experimental group. However, there was no significant change in the comparison between the groups in the WMFT and MAL QOM items (Table 2).

### 3.3. Corticospinal Excitability

In the comparison before and after the intervention within the groups, both the experimental and control groups showed significant changes in MEP amplitude. No significant change was observed in the comparison between the groups after the intervention. However, in comparing the amount of change before and after the intervention, a significant change was observed in the experimental group’s MEP amplitude (*p* = 0.001) (Table 2).

## 4. Discussion

Although many studies have confirmed the effect of AO and MI alone as cognitive interventions for severe stroke patients with limited voluntary movement, studies of a combination intervention of AO and MI are very rare [3,12,15,16]. Visual input ability for AO and internal imagination for MI may be difficult tasks for patients with severe stroke, and interventions may be limited depending on individual cognitive abilities [12]. However, a synergistic effect can be achieved if it acts as a complementary factor through parallel intervention in AO and MI. Therefore, this study aimed to investigate the effect of AO with MI intervention on upper extremity function and corticospinal activation in stroke patients.

In this study, the combined intervention of AO and MI showed a significant change in upper extremity function compared to AO alone. Both groups showed a significant improvement in FMA UE, WMFT, and MAL, which are upper extremity function evaluations. The difference in the amount of change between the groups was significant in FMA and MAL AOU in the experimental group. There was no significant difference in WMFT evaluation. The difficulty differs according to the detailed items of the three upper extremity function evaluation tools. Relatively, the WMFT evaluation tool has a large distribution of elaborate hand movements, so it is considered that the discriminative power of the subjects of this study for functional changes in patients with severe injuries is lowered [33]. Sun et al. (2016) applied AO + MI for four weeks to evaluate the recovery of ten stroke patients with hand motor dysfunction [18]. Simultaneous AO + MI FMA UE score improvement and strength improvement in tip pinch and mass grasp were reported. It has been demonstrated that applying AO + MI can improve upper extremity motor function after stroke. Aoyama et al. (2020) investigated the effect on hand control ability using AO + MI, targeting normal people. In the experimental group, the task of manipulation training in the hands with different difficulties was performed using AO + MI [24]. As a result, hand function improved in the experimental group that performed AO + MI, and better results were shown in the high-difficulty manipulation training task. As the evidence supporting the results of this study, it may be difficult to generate an internal motor image for actual functional activities only by inputting visual information when severe stroke patients performed AO only [34]. Furthermore, visual information and motor image generation were thought to be integrated using AO and MI, contributing to improving upper extremity movement ability. In addition, the upper extremity movements used for functional activities are complex and difficult with multi-step sequencing, however, the speed and accuracy could be improved using AO + MI [22].

Both the experimental group and the control group showed a significant level of change in the MEP amplitude test for corticospinal activation. However, in the comparison of the amount of MEP amplitude change between the two groups, there was a significant change in the experimental group. In a study by Macuga and Frey (2012) using fMRI, the activation of cortical regions was similar to that of active movements in normal subjects when AO + MI was used [35].

In a study that used fMRI in the general population, Taube et al. (2015) reported greater activation in the premotor area, supplementary motor area (SMA), basal ganglia, and cerebellum using AO + MI compared to AO and MI alone [36]. The results showed the activation of a network system between various areas for performing movements. Although the above studies used normal people, there is evidence that the combined intervention of AO and MI can show the same brain region activation as when the actual movement was performed in severe stroke patients with limited active movement. The results showing brain region activation can support the results of this study.

Eaves et al. (2016) reported a more pronounced electrophysiological activity for primary sensorimotor domains in the alpha and beta frequency bands for AO + MI in normal subjects using a quantitative electroencephalogram [37]. In another study, AO + MI showed greater corticospinal excitability than the single intervention using MEP amplitude measurement in healthy subjects [38]. It was suggested that the activity task of AO + MI was a functional activity using the fine movements of the hand selected by the subject and had a positive effect on the research results [39]. In this study, since the intervention was conducted by selecting five tasks out of ten using fine hand movements presented in the study, it may have affected the improvement of motivation, concentration, and corticospinal activation. In addition, since the two studies above used normal subjects, it can be considered a valuable result to present the evidence for a significant effect on patients with severe stroke in this study. Furthermore, since the participants selected the target tasks, participation in the intervention can be increased by focusing on the movement of personal meaning, thereby improving motor function by increasing attention, confidence, and self-efficacy.

This study has some limitations. First, the participants were acute stroke patients, and there is a possibility that it may be mixed with the effect of natural recovery. Therefore, selecting participants using a systematic classification of abilities will be necessary. Second, it is difficult to generalize the study results due to the small number of participants. Further studies on a larger stroke population should be conducted in the future.

## 5. Conclusions

The purpose of this study was to investigate the effect of MI combined with AO on corticospinal cord activation and upper extremity function in severe stroke patients. MI and AO are other forms of motion simulation that activate the motor system without physical activity. Previous studies of interventions with AO and MI alone reported positive effects, but this study tried to investigate the complementary synergistic effects of two interventions with different theoretical backgrounds. As a result of this study, AO with MI provided scientific evidence for the enhancement of upper extremity function and increased activation of the corticospinal cord in severe stroke patients with limited movement. Interventions accessible to home-based rehabilitation through education for stroke patients and caregivers have little economic cost. Therefore, they may be proposed as a particularly needed intervention in the era of the COVID-19 pandemic.

## Figures and Tables

**Table 1:** Characteristics of participants.

Characteristics	Experimental Group (*n* = 22)	Control Group (*n* = 23)	X^2^/t	*p*
Age (year), mean ± SD	62.68 ± 8.54	63.43 ± 9.57	−0.276	0.784
Gender (male/female)	12/10	12/11	−0.156	0.877
Type of stroke (Hemorrhage/Infarction)	9/13	11/12	0.457	0.650
Side of stroke (Right/Left)	12/10	7/16	−1.647	0.463
Time since onset of stroke months, mean ± SD	4.82 ± 2.08	4.61 ± 1.69	0.370	0.713

SD: standard deviation.

**Table 2:** Comparison of results between the experimental group and control group.

	Experimental Group				Control Group				Between Groups *p*-Values
	Before Treatment	After Treatment	Mean Difference	*p*-Value	Before Treatment	After Treatment	Mean Difference	*p*-Value	
FMA UE	15.50 (3.41)	18.59 (3.86)	3.09 (2.82) ^††^	0.000 **	13.04 (5.02)	14.22 (5.06)	1.17 (1.07)	0.000 **	0.002 ^†^
WMFT	12.09 (4.40)	14.41 (4.95)	2.32 (1.70)	0.000 **	11.52 (4.83)	13.00 (5.03)	1.00 (1.20)	0.000 **	0.350
MAL QOM	0.89 (0.40)	1.62 (0.73)	0.72 (0.54)	0.000 **	1.07 (0.33)	1.66 (0.76)	0.59 (0.60)	0.000 **	0.834
MAL AOU	0.84 (0.32)	2.02 (0.48)	1.18 (0.45) ^†^	0.000 **	1.00 (0.37)	1.60 (0.68)	0.59 (0.57)	0.000 **	0.022 ^†^
MEP amplitude	84.29 (34.43)	128.90 (49.82)	44.60 (47.51) ^†^	0.000 **	101.66 (46.01)	109.88 (46.86)	8.21 (6.97)	0.000 **	0.194

The values are mean (±standard deviation); FMA UE: Fugl–Meyer Assessment for Upper Extremity; WMFT: Wolf Motor Function Test; MAL AOU: Motor Activity Log Amount of Use; MAL QOM: Motor Activity Log Quality of Movement; MEP: Motor Evoked Potential, The values are mean ± standard deviation, ** *p* < 0.001 by paired *t* test, ^†^ *p* < 0.05, ^††^ *p* < 0.001 by independent *t* test.

## Data Availability

The datasets generated during the current study are available from the corresponding author upon reasonable request.
